# Supplementary material for: Modeling the roles of cohesotaxis, cell-intercalation, and tissue geometry in collective cell migration of Xenopus mesendoderm
Source: Biol Open. 2024 Aug 19;13(8):bio060615. doi: 10.1242/bio.060615 (PMC11360141; doi:10.1242/bio.060615)
Supplement: Supplementary information [file biolopen-13-060615-s1.pdf]

Table S1. Mesendoderm migration in standard DFA with 1.0 mM Ca++ vs. DFA with 0.8 mM Ca++. DMZ explant migration in reduced Ca++ media

	DFA with 1.0 mM Ca++ (um/hr; n = 12, from 4 clutches)	DFA with 0.8 mM Ca++ (um/hr; n = 11, from 4 clutches)
1 hr	113	139*
2 hr	110	126

Mean rate of migration over first 1 hour or first 2 hours in standard DFA media vs. DFA with 0.8 mM Ca++ and Mg++. Migration rate during the first hour was significantly different (* $p = 0.044$) between standard and Ca++ reduced DFA by two-way ANOVA. See Materials and Methods for further details.

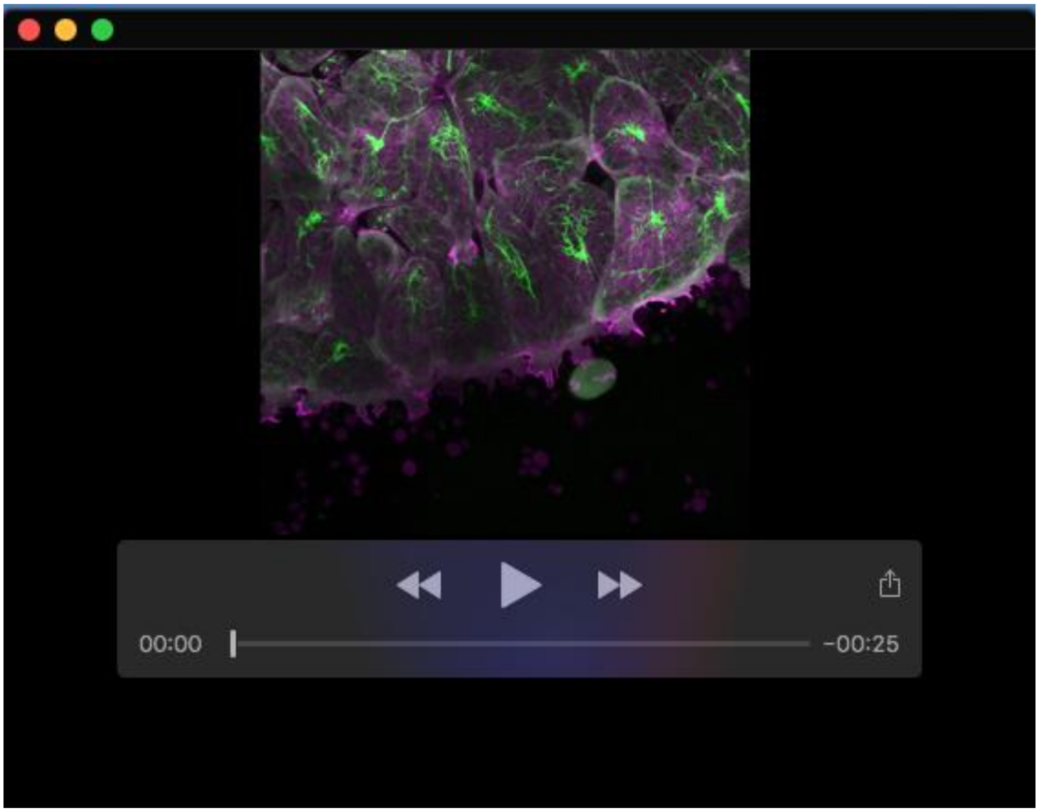

Movie 1. Timelapse movie of single DMZ explant on fibronectin. Actin (magenta) and Keratin (green) were labeled by injection of mRNA transcript encoding LifeAct-RFP (actin) and XCK-GFP (cytokeratin filaments). Confocal Z-stacks were collected at 2 min intervals (15 frames).

Biology Open • Supplementary information

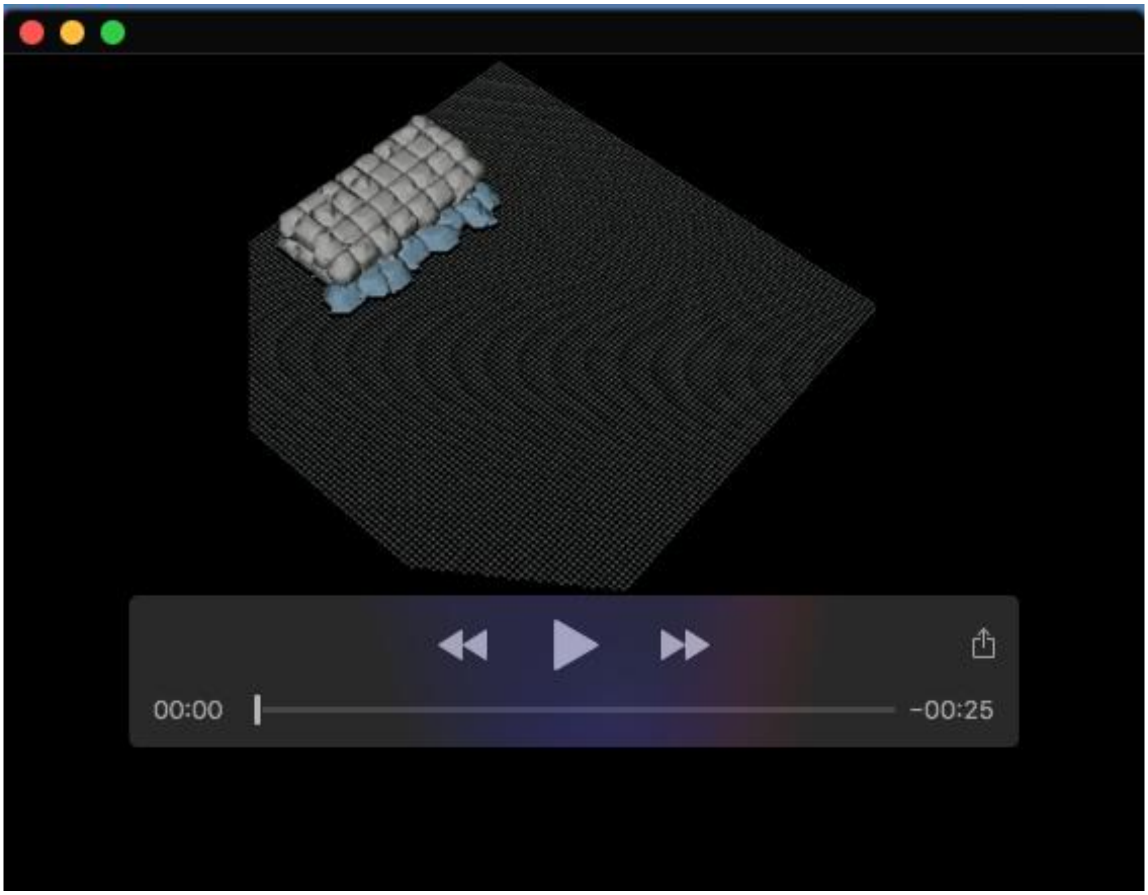

Movie 2. Timelapse sequence of a simulated single DMZ explant migrating on a substrate.

Biology Open • Supplementary information

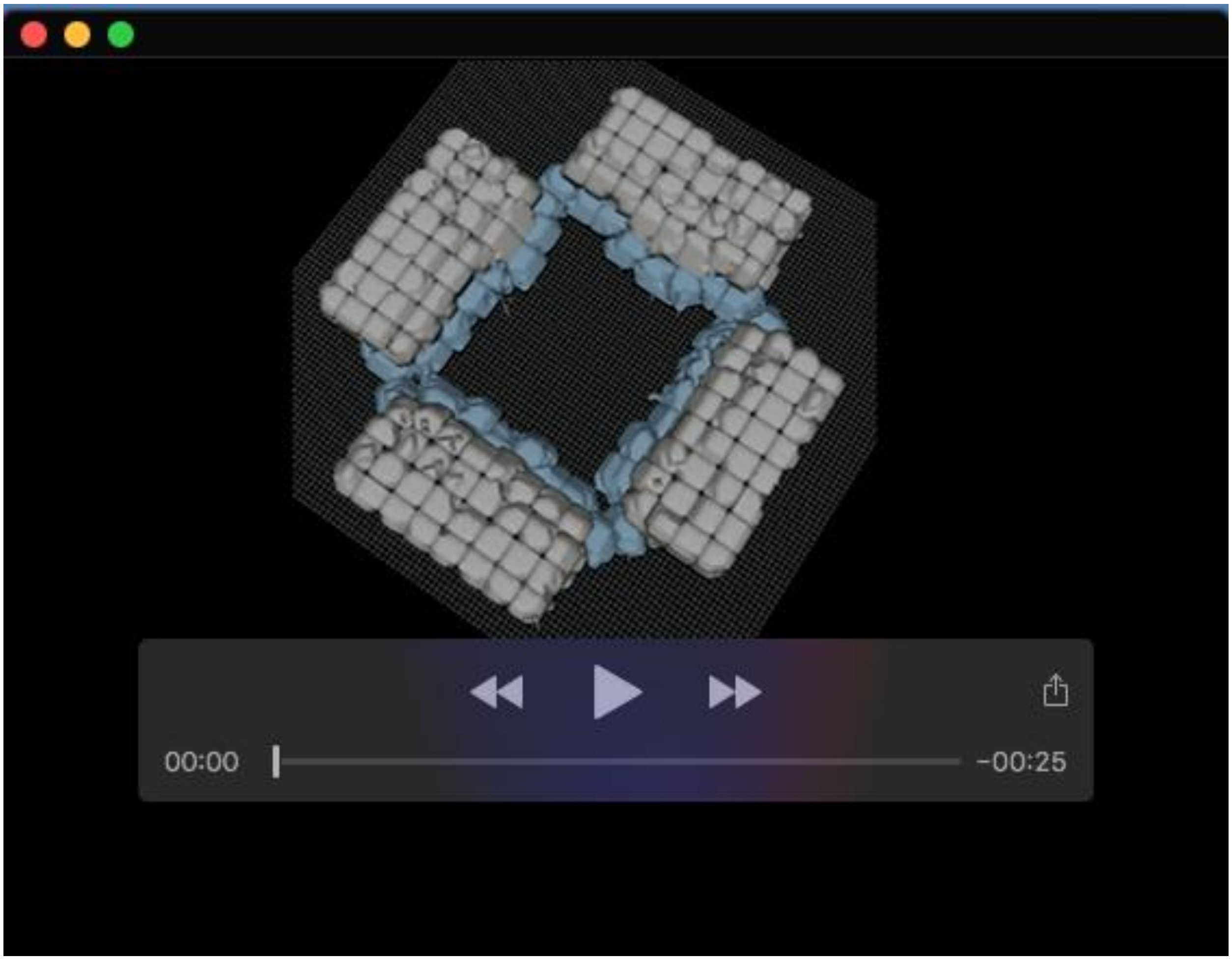

Movie 3. Timelapse sequence of four simulated DMZ explants in the ITR configuration

Biology Open • Supplementary information

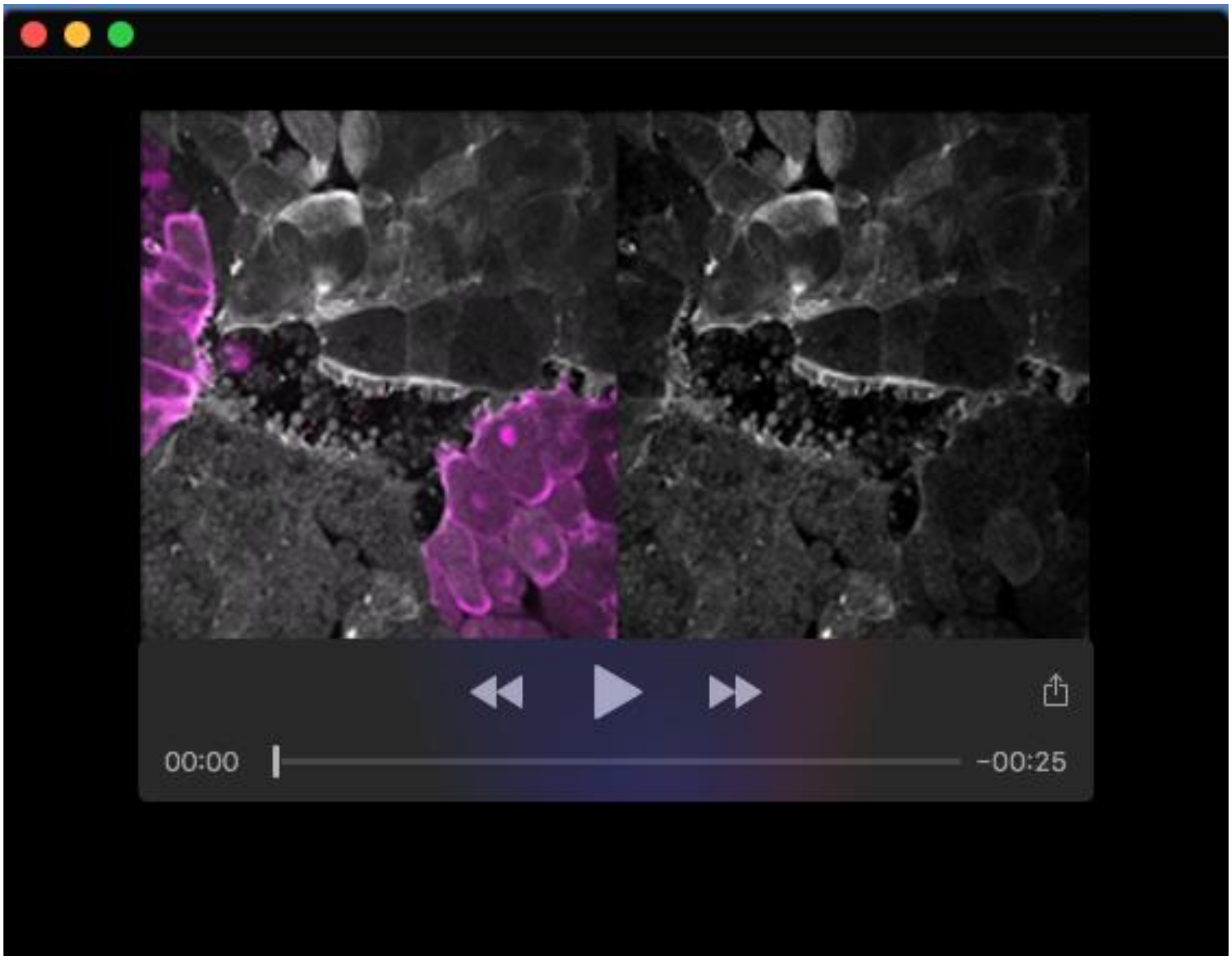

Movie 4. Four DMZs migrating on a fibronectin coated substrate in the round (ITR). Actin (greyscale) was labeled by injection of mRNA encoding LifeAct-RFP. Two of the four DMZs were labeled with Alexa 488 dextran (magenta-left panel) to identify individual DMZs. Confocal Z-stacks were collected at 2 min intervals (25 frames). The debris in the center of the 4 DMZs is yolk granules released from the cells during microdissection.

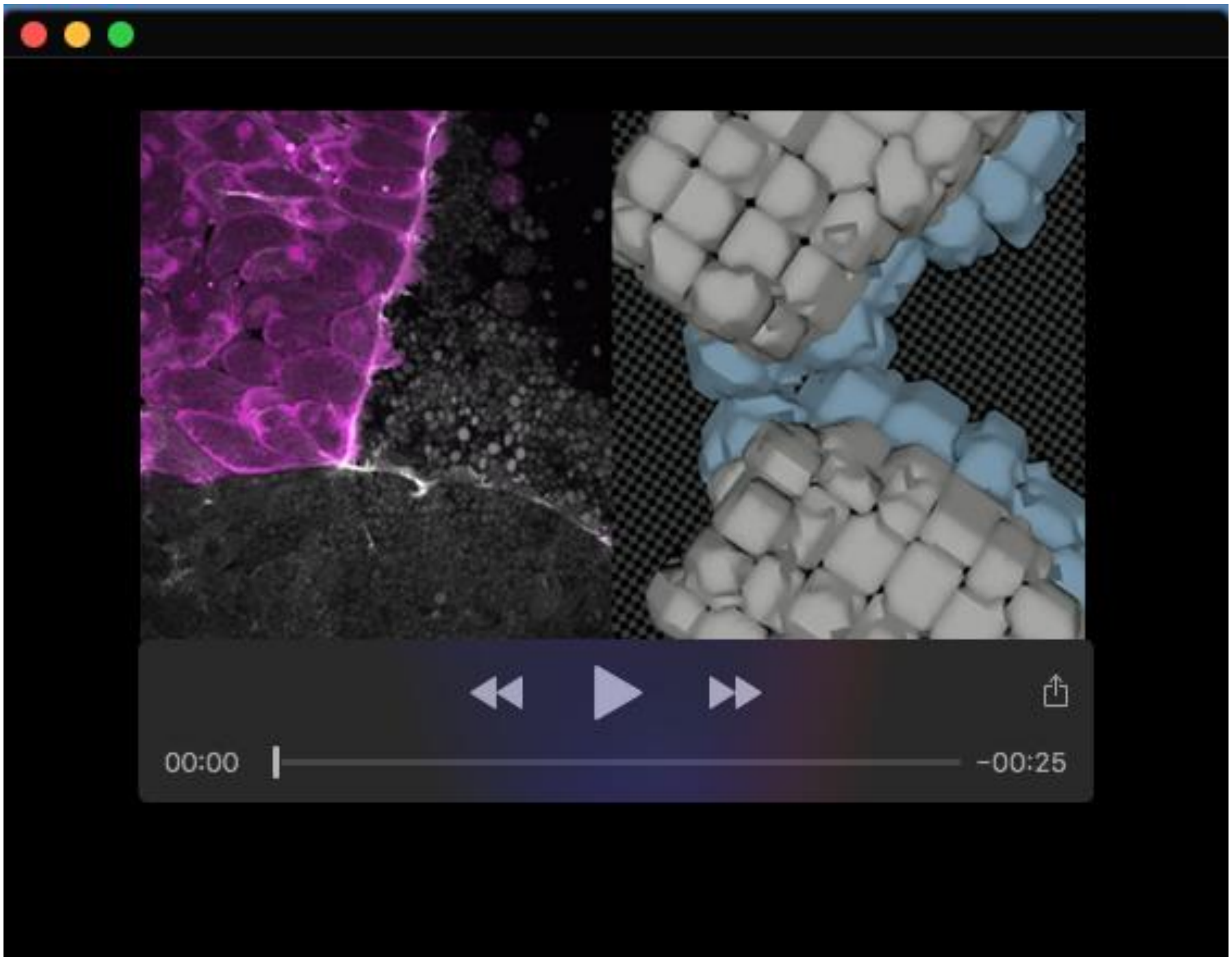

Movie 5. Interface between two DMZs *in vitro* (left) and *in silico* (right) migrating from 4 DMZs placed ITR. Left: Actin (greyscale) was labeled by injection of mRNA encoding LifeAct-RFP. Two of the four DMZs were labeled with Alexa 488 dextran (magenta-left panel) to identify individual DMZs. Confocal Z-stacks were collected at 3 min intervals (11 frames). Representative frames from a ITR simulation are shown on the right.

Biology Open • Supplementary information

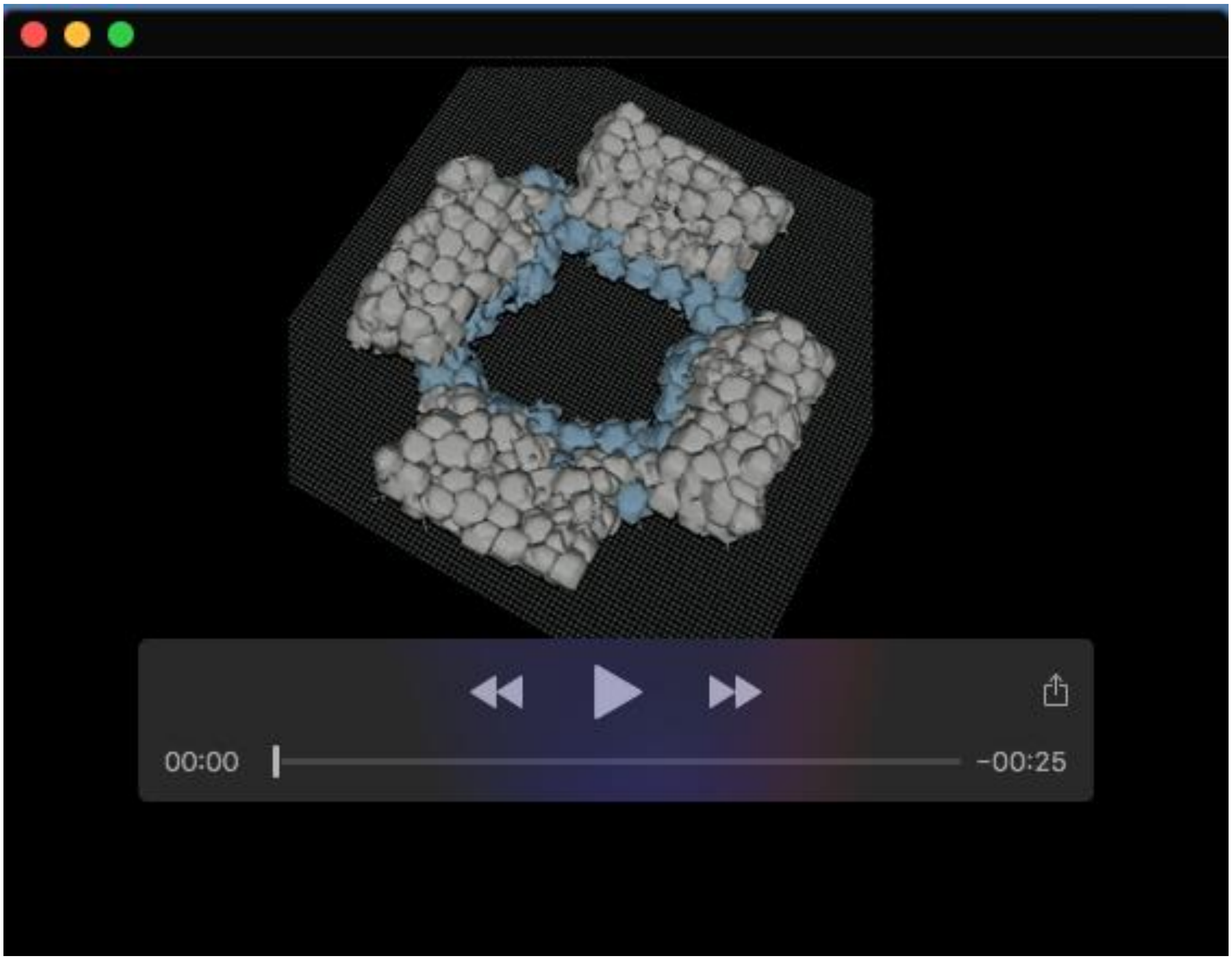

Movie 6. Timelapse sequence of four simulated DMZ explants in the ITR configuration with cell intercalation allowed.

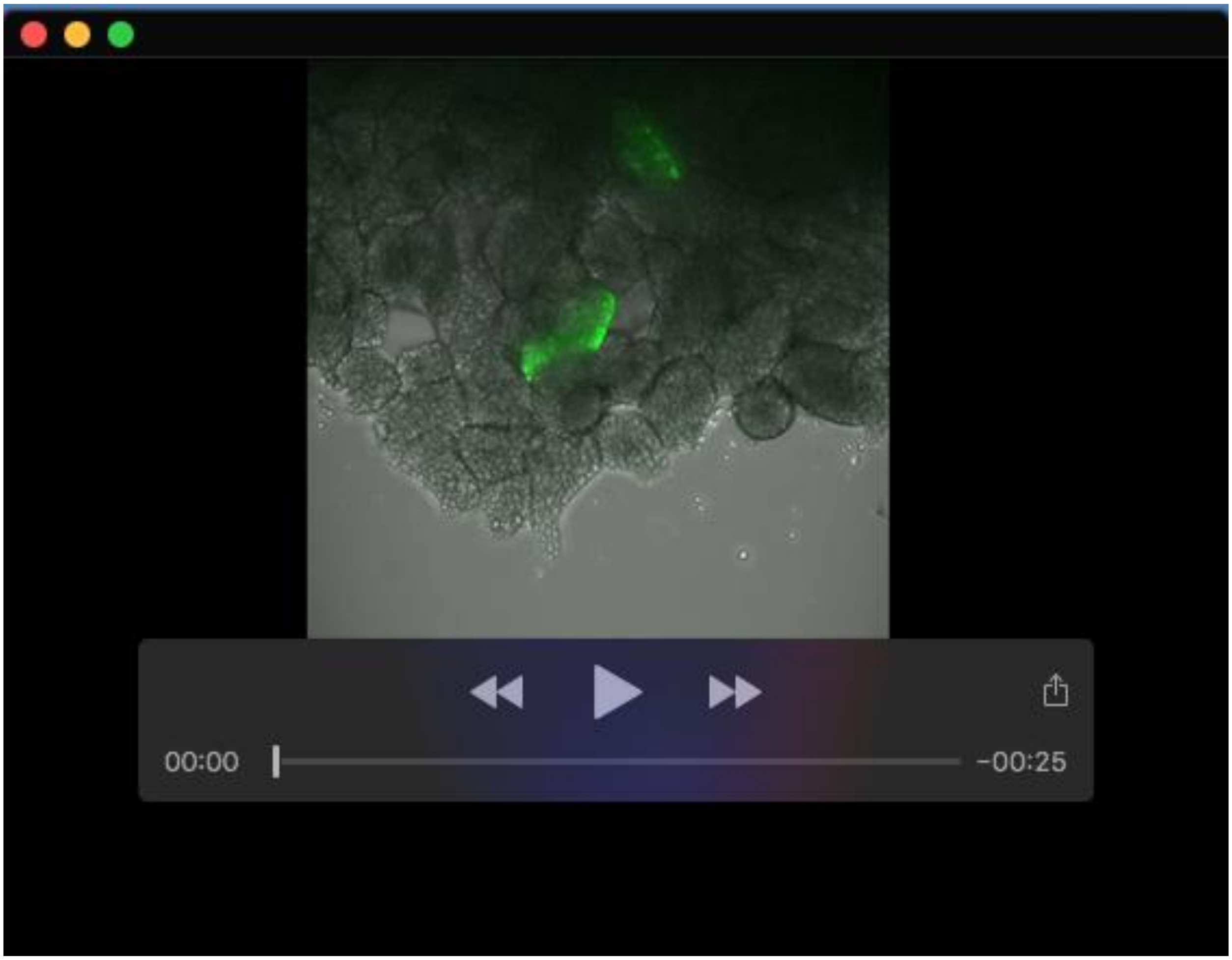

Movie 7. Timelapse movie showing integration of single dextran Alexa488-labeled cells applied onto an unlabeled DMZ explant migrating on fibronectin substrate. Mesendoderm cells, labeled by injection of Alexa488 dextran (green), were dissociated from sibling embryos and placed on top of an unlabeled DMZ migrating on a fibronectin substrate. Confocal Z-stacks were collected at 1 min intervals (32 frames).

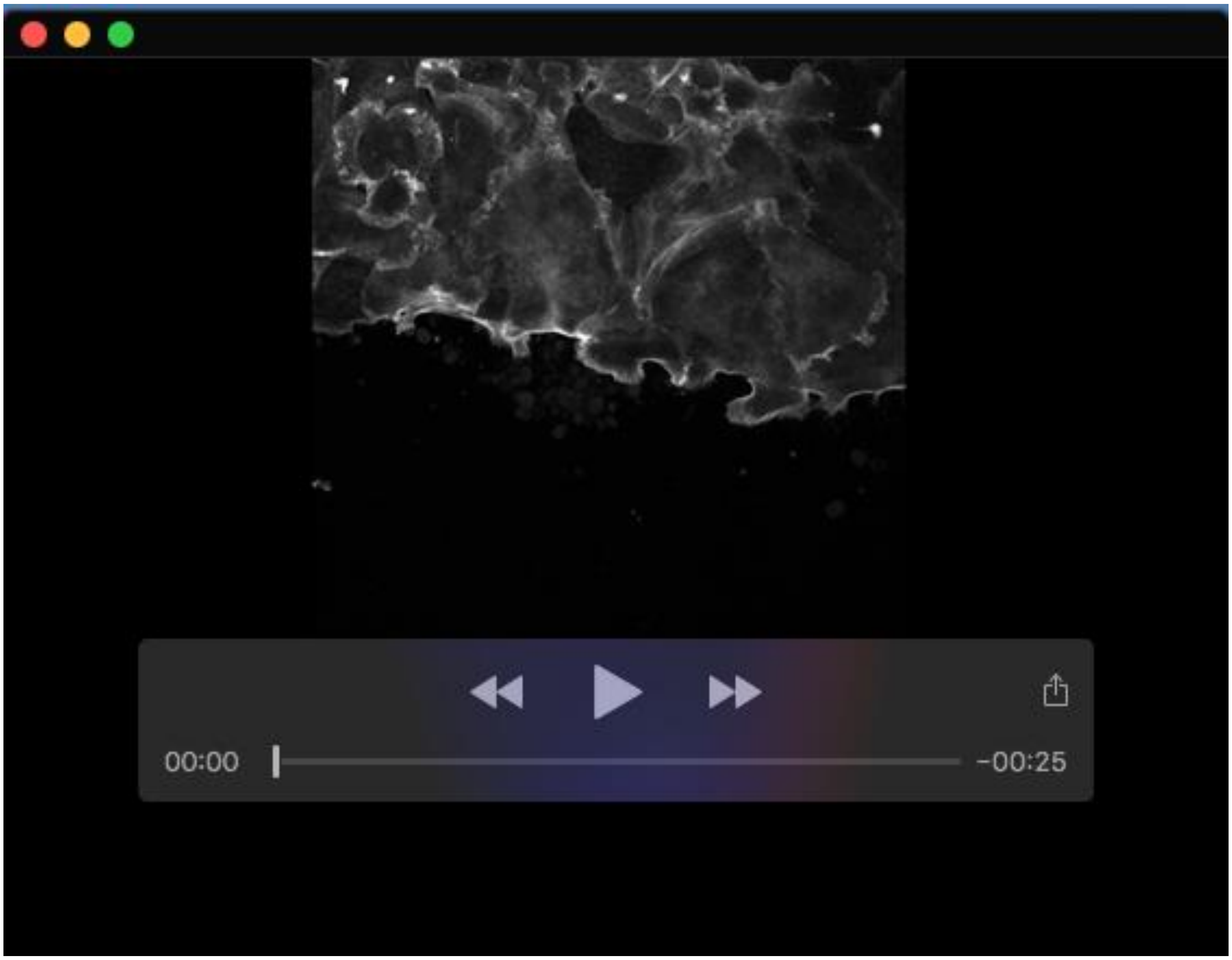

Movie 8. Timelapse movie of single DMZ explant on fibronectin with intercalating cell. Actin (greyscale) was labeled by injection of mRNA encoding LIfeAct-RFP. An intercalating cell is indicated by the yellow arrow. Confocal Z-stacks were collected at 2 minute intervals (17 frames).
